# Determinants and Functions of CAFs Secretome During Cancer Progression and Therapy

**DOI:** 10.3389/fcell.2020.621070

**Published:** 2021-01-22

**Authors:** Jenniffer Linares, Juan A. Marín-Jiménez, Jordi Badia-Ramentol, Alexandre Calon

**Affiliations:** ^1^Cancer Research Program, Hospital del Mar Medical Research Institute (IMIM), Barcelona, Spain; ^2^Department of Medical Oncology, Catalan Institute of Oncology (ICO) - L'Hospitalet de Llobregat, Barcelona, Spain

**Keywords:** cancer-associated fibroblast (CAF), secretome, secreted factors, growth factors, cancer, tumor microenvironment (TME), metastasis, therapy

## Abstract

Multiple lines of evidence are indicating that cancer development and malignant progression are not exclusively epithelial cancer cell-autonomous processes but may also depend on crosstalk with the surrounding tumor microenvironment (TME). Cancer-associated fibroblasts (CAFs) are abundantly represented in the TME and are continuously interacting with cancer cells. CAFs are regulating key mechanisms during progression to metastasis and response to treatment by enhancing cancer cells survival and aggressiveness. The latest advances in CAFs biology are pointing to CAFs-secreted factors as druggable targets and companion tools for cancer diagnosis and prognosis. Especially, extensive research conducted in the recent years has underscored the potential of several cytokines as actionable biomarkers that are currently evaluated in the clinical setting. In this review, we explore the current understanding of CAFs secretome determinants and functions to discuss their clinical implication in oncology.

## Introduction

Cancer-associated fibroblasts (CAFs) are contributing to the production of a wide variety of secreted factors impacting tumor progression by directly regulating malignant cancer cells aggressiveness or by indirectly reprogramming tumor immunity and angiogenesis (Sahai et al., 2020). Hence, molecular and functional inter- and intra-tumoral heterogeneity of CAFs has been a recent focus in oncology research. It is widely accepted that the functional phenotype of CAFs is in part determined by the cell of origin, including but not restricted to local resident fibroblasts (D'Arcangelo et al., 2020; Sahai et al., 2020). An alternative hypothesis advocating for CAF being a cell state depending on autocrine and paracrine signaling rather than a cell type has also been proposed (Kalluri, 2016). Whether CAFs functional heterogeneity is maintained among different solid tumor types or is a constant evolutionary state is still a debated question. However, recent studies have been investigating the determinants of CAFs secretome and their therapeutic interest across different tumor types. Molecular biomarkers predicting the risk of relapse and the potential benefit from treatments are currently needed for clinical decision-making. In an attempt to reach a more comprehensive evaluation of tumors, many CAFs-secreted factors have been included in gene expression signatures that are considered suitable prognostic tools for clinical diagnosis and prognostication (Berdiel-Acer et al., 2014; Karlan et al., 2014; Yue et al., 2019). As a matter of fact, recent advances in the understanding of CAFs secretome determinants and functions have brought to light the multiple benefits of using CAFs-secreted factors as actionable biomarkers for cancer diagnosis, treatment, and prognosis.

## Determinants of CAFS Secretome

### Crosstalk With Tumor Cells and TME Components

#### Autocrine- and Paracrine-Secreted Factors

**Figure 1:**
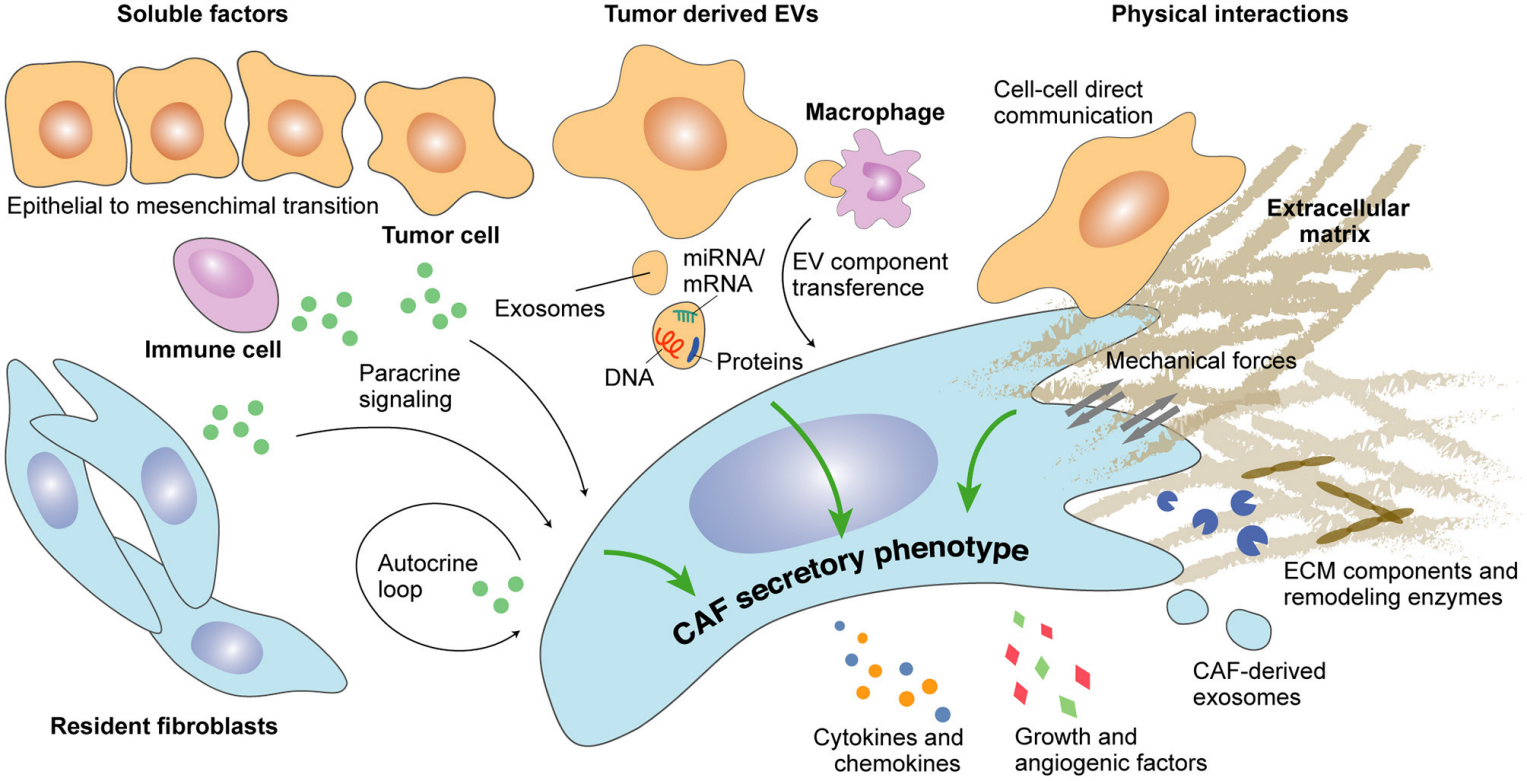
Determinants of CAFs secretome during cancer progression. Secreted factors as well as extracellular vesicle (EV)-dependent autocrine and paracrine crosstalk modulate the secretory profile of CAFs. Direct physical interaction between CAFs and epithelial cancer cells or between CAFs and components of the extracellular matrix may additionally regulate CAFs secretome.

Multiple autocrine loops impacting CAFs secretome have been discovered in the recent years (Figure 1). Among them, members of the transforming growth factor beta (TGF-beta) superfamily are known to be the main inducers of CAFs activation. Of note, CAFs secrete large amounts of TGF-beta isoforms 1, 2, and 3 (reviewed in Kalluri, 2016). In turn, secreted TGF-beta maintains a self-sustained active state -typically regarded as myofibroblasts- characterized by alpha smooth muscle actin (alpha-SMA) expression (Orimo et al., 2005; Kojima et al., 2010). Other members of the TGF-beta superfamily, such as Nodal, induce pro-tumorigenic phenotypes in fibroblasts from melanoma and colorectal cancer (CRC) (Li et al., 2019). Likewise, activin A has showed the ability to induce a secretory phenotype in CAFs via the SMAD-2-mediated transcriptional regulation of genes encoding extracellular matrix (ECM) components, ECM regulators, and soluble factors (Cangkrama et al., 2020). Alternatively, CAFs secretome may be maintained during tumor progression through enhanced stromal cell-derived factor (SDF)-1 autocrine signaling loops and increased co-expression of receptors, such as TGF-beta-RI and CXC receptor (CXCR)-4 (Kojima et al., 2010). In this sense, Scherz-Shouval et al. reported an increased production of TGF-beta-2 and SDF-1 factors by CAFs upon heat shock factor (HSF)-1 cytoplasmic translocation to the nucleus (Scherz-Shouval et al., 2014). HSF-1 is a transcription factor that mediates the cellular response to different types of stress, such as hypoxia or proteotoxic stress (Dayalan Naidu and Dinkova-Kostova, 2017).

Complex paracrine signaling through cancer cell-secreted factors also regulates CAFs secretome during tumor progression (Figure 1) and may depend on genetic alterations occurring in cancer cells. Indeed, KRAS mutant but not KRAS wild-type pancreatic ductal adenocarcinoma (PDAC) cancer cells induce CAFs activation through CXCR-2, leading to a nuclear factor-kappa B (NF-κB)-mediated secretion of pro-tumoral cytokines interleukin (IL)-4, IL-10, and IL-13 (Awaji et al., 2020). Several additional studies have reported a pro-tumorigenic crosstalk between cancer cells and CAFs. For instance, kallikrein (KLK)-4 produced by malignant and premalignant prostate lesions can act on normal fibroblasts through the activation of protease-activated receptor (PAR)-1, which leads to a CAF pro-angiogenic secretory phenotype characterized by the increased expression of Dickkopf-related protein 1 (DKK-1), growth differentiation factor 15 (GDF-15), hepatocyte growth factor (HGF), IL-8, and vascular endothelial growth factor (VEGF) and the decreased expression of insulin-like growth factor-binding protein (IGFBP)-3, monocyte chemoattractant protein 1 (MCP-1), and platelet-derived growth factor (PDGF)-AA (Kryza et al., 2017). In PDAC, S100 calcium-binding protein A11 (S100A11) secreted by cancer cells activates the surrounding fibroblasts through the S100A11–receptor for advanced glycation end products (RAGE)–tumor progression locus 2 (TPL2)–cyclooxygenase 2 (COX2) pathway to promote metastatic progression (Mitsui et al., 2019).

CAFs activation may also occur through paracrine signaling with non-cancer cells. For example, cytokines produced by immune cells during the inflammatory process may instruct CAFs function. Among them, IL-1β produced by immune cells in early hyperplastic lesions activates normal fibroblasts to become CAFs *via* the NF-κB pathway (Erez et al., 2010). Alternatively, granulin secretion by macrophages activates resident fibroblasts into tumor-promoting myofibroblasts sustaining metastatic growth in PDAC (Nielsen et al., 2016).

In addition to local intra-tumoral autocrine and paracrine signaling, systemic signaling involving steroid hormones, such as estrogens and androgens, is also able to modulate CAFs secretome, through binding to their receptors expressed in CAFs (Clocchiatti et al., 2018; Rothenberger et al., 2018). For instance, estrogens (E2) regulate the expression of several microRNAs (miRNAs) in breast cancer (BC)-derived CAFs (Vivacqua et al., 2019). In gastric cancer, estrogens stimulate CAFs secretion of IL-6, thereby promoting signal transducer and activator of transcription (STAT)-3 pathway-dependent cancer cells proliferation and invasion (Zhang Y. et al., 2020). Similarly, in prostate cancer (PCa), the activation of CAFs–androgen receptor (AR) with dihydrotestosterone modulates the secretion of pro-tumorigenic factors impacting cancer cell growth (Tanner et al., 2011). Conversely, AR blockade in CAFs decreases the expression of pro-tumorigenic factors, such as insulin-like growth factor (IGF)-1, fibroblast growth factor (FGF)-7, FGF-10, SDF-1, HGF, and TGF-beta 2 (Yu et al., 2013).

As illustrated by the opposite gene expression programs regulated upon FGF and TGF-beta pathway activation (Bordignon et al., 2019), CAFs secretome heterogeneity may be the result of a delicate balance between autocrine and paracrine signaling stimulated simultaneously and activating CAFs in different ways. It is worth noting that autocrine and paracrine triggering cues educating CAFs secretory functions may be tumor-dependent or even cancer subtype-dependent. Thus, the identification of multiple CAFs activation and secretion programs could greatly improve current molecular classification of cancer.

#### Extracellular Vesicles

Extracellular vesicles (EVs) are spherical membrane formations comprising exosomes and microvesicles, which can carry different molecules, such as proteins, DNAs, non-coding RNAs, and miRNAs/mRNAs. While both are playing a key role in distant intercellular communication, exosomes are derived from the endosomal system, and microvesicles are produced by the plasma membrane (reviewed in van Niel et al., 2018). EV-based intercellular communication between cancer cells and the tumor microenvironment (TME)–including CAFs (Figure 1)–promotes cancer progression in multiple ways (reviewed in Han et al., 2019); however, EVs' ability to induce a secretory phenotype in CAFs still remains an open field of research (Webber et al., 2015). In some cases, tumor cell-derived EVs contain typical mitogenic factors, such as TGF-beta, which in ovarian cancer induces a pro-tumoral secretome leading to increased proliferation, motility, and invasiveness of ovarian cancer cells (Giusti et al., 2018). Furthermore, an increasing body of evidence points to the importance of EV-derived miRNA. For instance, in gastric cancer, EVs containing miR155, miR193b, and miR210 prime CAFs to secrete inflammatory chemokines, such as CXC ligand (CXCL)-1 and CXCL-8, through the activation of the Janus kinase (JAK)/STAT and NF-κB signaling pathways (Naito et al., 2019). Similarly, NF-κB activation in CAFs by EV-derived miR-1247-3p in metastatic hepatocellular carcinoma (HCC) leads to a tumor-promoting secretion of IL-6 and IL-8 (Fang et al., 2018). Alternatively, EV-derived miR-210 and miR-155-5p induce a pro-angiogenic switch in CAFs through the activation of the JAK-2/STAT-3 pathway in lung cancer and melanoma (Zhou et al., 2018; Fan et al., 2020). Additional signaling pathways have been associated with miRNA-driven secretory stimulation. For instance, EVs containing miR-10b contribute to the enhanced TGF-beta expression in CAFs through the phosphoinositide 3-kinase (PI3K)/protein kinase B (AKT)/mammalian target of rapamycin (mTOR) signaling pathway in CRC (Dai et al., 2018). Finally, EVs containing coding mRNA have also been reported to trigger a secretory phenotype in CAFs. In this sense, cancer cell-derived EVs containing mRNA coding for CXCR-4 and IGF-1R promote CAFs secretion of growth factors, such as VEGF in acute myeloid leukemia (Huan et al., 2013).

Interestingly, EV-induced CAFs may in turn shed additional EVs that will further support tumor growth by conferring enhanced proliferative and survival capabilities to cancer cells, thus establishing an EV-mediated bidirectional intercommunication (Savardashtaki et al., 2019; Yang et al., 2019). For instance, CAFs exposed to cancer cell-derived EVs in Hodgkin lymphoma are primed to secrete EVs together with pro-inflammatory cytokines and angiogenic factors (Dörsam et al., 2018). Similar to cancer cell-derived EVs, CAF-derived EVs contain commonly miRNA, which has been described to promote migration and resistance to treatment in several tumors (Dourado et al., 2019; Hu et al., 2019; Qin et al., 2019; Sun et al., 2019; Wang J.-W. et al., 2019). Besides miRNA, CAF-derived EVs containing mitochondrial mRNA can educate cancer cells to increase oxidative phosphorylation system (OXPHOS) metabolism, which consequently induces escape from dormancy (Sansone et al., 2017).

EV-mediated activation of CAFs can also occur indirectly through stromal cell mediators present in the TME. Indeed, a recent study showed that tumor-associated macrophages incorporate and transfer cancer cell-derived EVs to CAFs, which allows the formation of a pro-tumorigenic microenvironment (Umakoshi et al., 2019).

Moreover, EVs content may be changing during tumor progression, which could result in a temporal modulation of CAFs secretory phenotypes. Supporting this notion, EVs derived from primary or metastatic CRC promote different CAF functional profiles, switching from a pro-angiogenic to a more ECM remodeling phenotype (Rai et al., 2019). Therefore, EV-mediated communication between tumor cells and CAFs may depend on alterations in the composition of secreted EVs during tumor progression. However, additional research is needed to confirm this hypothesis.

#### Cell–Cell Contact and Mechanical Interactions

CAFs/cancer cells physical interactions can modulate CAFs functions (Yamaguchi and Sakai, 2015), and the high complexity of this interplay has been recently illustrated (Arwert et al., 2020). For instance, CAFs may sense cancer cell genomic stress through cytoplasmic transcytosis and respond by expressing interferon (IFN)-β1, leading to an increased production of chemokines, cytokines, and other inflammatory factors [CXCL-1 and 10, granulocyte-macrophage colony-stimulating factor (GM-CSF), macrophage migration inhibitory factor (MIF), IL-6, and IL-8, among others] (Arwert et al., 2020). Hetero-cellular gap junction communications have also been described between CAFs and cancer cells. This mechanism promoting epithelial-to-mesenchymal transition (EMT), migration, and invasiveness appears to be rather unidirectional—from CAFs toward cancer cell (Luo et al., 2018); however, a better understanding of the underlying molecular mechanisms is still required.

Mechano-sensing between CAFs and the surrounding ECM may equally modulate CAFs secretome. For example, matrix stiffness and contractile forces have been shown to determine CAFs behavior through different mechano-sensitive pathways. Among them, non-canonical YAP pathway activation is promoting CAFs ECM remodeling and angiogenic functions (Calvo et al., 2013). Indeed, secretion of pro-angiogenic VEGF-A by CAFs depends on factors involved in YAP pathway activation (Calvo et al., 2015).

These findings suggest a significant role of physical interactions in determining CAFs secretory phenotype. Remarkably, spatial (invasive front or tumor core) as well as temporal (early or late stage cancer) characteristics may influence mechano-sensing and should be considered when studying forces instructing CAFs secretory functions (Acerbi et al., 2015; Bauer et al., 2020).

### Spatial and Temporal Plasticity

**Figure 2:**
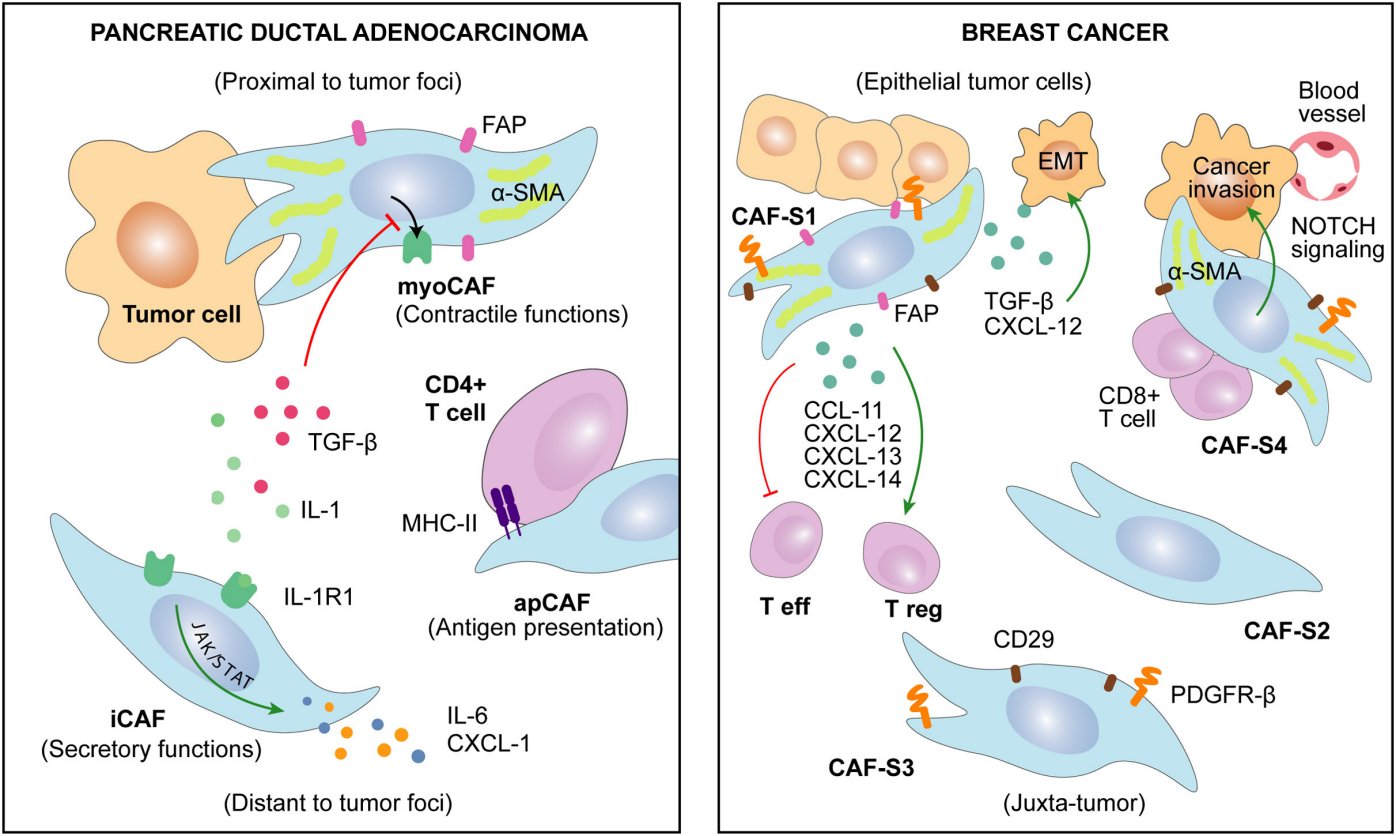
Several CAFs subtypes have been recently described in cancers of distinct origin. CAFs heterogeneity is associated with specific markers and discrete functions. Remarkably, CAFs characteristics may be related to their spatial localization in the tumor.

Intra-tumor heterogeneity of CAFs functional subtypes and their differential spatial pattern are already a widespread knowledge (Lambrechts et al., 2018; Awaji and Singh, 2019; Neuzillet et al., 2019). Thus, an effect of CAFs intra-tumoral location upon their secretory function may be expected. Remarkably, fibroblasts located next to the invasive front of BC show higher capacity to induce cancer cells migration and EMT in comparison with those located in the epicenter of the tumor (Gao et al., 2010). In pancreatic cancer, two spatially separated subtypes were identified (Figure 2). An inflammatory CAFs subtype (iCAFs), distant from cancer cells, showed a secretory phenotype with high interleukin and chemokine production [leukemia inhibitory factor (LIF), IL-6, IL-11, IL-1, CXCL-1] in comparison with periglandular myofibroblastic CAFs (myoCAFs), specialized in stromal remodeling functions (Öhlund et al., 2017). However, these two subtypes may be the borders of a functional spectrum depending on IL-1-R1 expression, conditioned by the balance between TGF-beta (pro-myoCAFs) and IL-1/JAK/STAT (pro-iCAFs) signaling activation (Biffi et al., 2019). An additional antigen-presenting CAFs (apCAFs) population activating CD4+ T cells was recently identified in PDAC (Figure 2), suggesting CAFs subtype-specific immunomodulatory capacity (Elyada et al., 2019). Along this line, Neuzillet and colleagues highlighted the complexity of CAFs heterogeneity in PDAC, describing at least four different functional and spatially distributed subtypes depending on periostin (POSTN), myosin 11 (MYH-11), and podoplanin (PDPN) expression (Neuzillet et al., 2019). Similarly, in BC (Figure 2), a subset of myofibroblastic CAFs (CAF-S1: CD-29^Med^ FAP^Hi^ FSP-1^Low−Hi^ αSMA^Hi^ PDGFRb^Med−Hi^ CAV-1^Low^) secreting differentially higher amount of CCL-11, CXCL-12, CXCL-13, and CXCL-14 was predominantly detected close to epithelial tumor cells (Costa et al., 2018). The other myofibroblastic (αSMA^Hi^) CAF-S4 subtype was preferentially located within the tumor tissue, whereas CAF-S3 (FSP-1^Med^ and PDGFRb^Med−Hi^) was detected in the juxta tumoral healthy tissue. CAF-S2 subtype (low or negative expression of stromal markers) appeared to be equally distributed in both areas (Costa et al., 2018).

For greater complexity, temporal evolution of CAFs during tumor progression makes it difficult to assess the exclusive effect of spatial distribution (Kalluri, 2016; D'Arcangelo et al., 2020). Indeed, Nidogen-2, a protein secreted by “vascular CAFs” in murine BC model, was firstly detected among perivascular cells but relocated within the tumor stroma at later stages of tumor progression (Bartoschek et al., 2018). In PDAC patients, different phenotypes were detected by CAFs secretome analysis of primary tumors and matched metastatic tissue (Öhlund et al., 2017). In addition, the above-mentioned S1 and S4 CAFs subsets have been recently found to be enriched in metastatic BC lymph nodes in comparison with matched primary tumor tissue (Costa et al., 2018; Pelon et al., 2020). In this setting, CXCL-12 secretion was found to be responsible of CAF-S1 tumor-promoting phenotype, whereas CAF-S4 seemed to be specialized in NOTCH-dependent contractile and remodeling functions (Pelon et al., 2020). Similarly, CAFs isolated from prostate tumors at different stages revealed that CAFs secretome evolves during PCa development. While localized PCa-derived CAFs are characterized by FGF-7 secretion, CAFs from metastatic PCa showed increased levels of matrix metallopeptidase (MMP)-11 and heat shock 70 kDa protein 1A (HspA1A) (Eiro et al., 2017).

As previously mentioned, increasing evidence suggests that PCa-derived CAFs secretome is modulated by AR signaling (Yu et al., 2013; Cioni et al., 2018). Interestingly, AR expression by CAFs evolves during PCa progression (Olapade-Olaopa et al., 1999; Gevaert et al., 2018). Indeed, metastatic PCa and castration resistance PCa display significantly lower stromal AR expression than localize tumor- and androgen-dependent PCa (Li et al., 2008; Singh et al., 2014). These observations suggest that a temporal regulation of CAFs secretome may occur through the modulation of hormone receptors expression during cancer progression.

Overall, a complex relationship between temporal and spatial influence may instruct CAFs secretome. However, it is still unknown whether tumor progression orchestrates CAFs function and plasticity according to spatial factors or, conversely, distinct CAFs spatial subtypes are modeling tumor dynamics.

### Anti-cancer Therapy as a Determinant of CAFs Secretome

#### Chemotherapy

**Figure 3:**
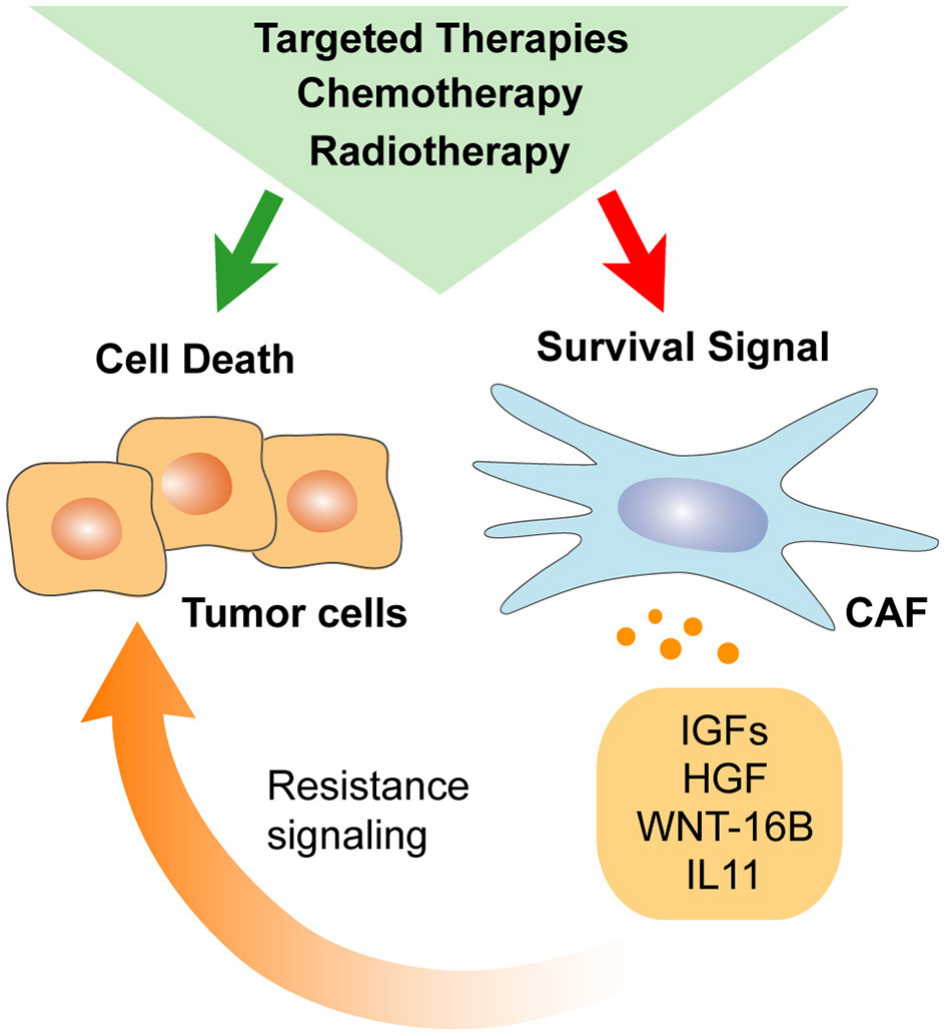
Anti-cancer therapy impacts CAFs secretome. Stromal elements develop their own response to systemic chemotherapy, radiotherapy, and targeted therapy. As a result, CAFs response to treatment may diminish direct anti-cancer drug efficiency by promoting cancer cells self-renewal and survival.

Several studies have addressed the modulatory effect of standard chemotherapy (CT) on the TME. CT induces shrinkage of cancer cells compartment resulting in an increased representation of stromal cells in residual tumors (Goto et al., 2017). In this context, factors secreted by CAFs after CT may significantly enhance tumor regrowth from residual cancer cells (Hisamitsu et al., 2019). Current evidence points to CT-induced DNA damage being a key mechanism influencing the repertoire of CAF-secreted factors after treatment (Figure 3). Indeed, CAFs support tumor regrowth after DNA damage-mediated NF-κB signaling activation through secretion of WNT-16B (Sun et al., 2012). Alternatively, treatment of lung adenocarcinoma with cisplatin enhances IL-11 secretion by CAFs, which in turn promotes resistance of cancer cells to CT through the STAT-3 signaling pathway (Tao et al., 2016). Of note, CAF-induced IL-11/STAT-3 cell–cell survival signaling has been reported in other tumor types (Calon et al., 2012). Thus, CAF-dependent IL-11 resistance mechanism may be of potential relevance in cancers of distinct origin. Similarly, a direct effect of gemcitabine and 5-FU regimen toward a tumor-supportive secretory phenotype of CAFs has been described in PDAC. In that case, higher expression of secreted factors [intercellular adhesion molecule 1 (ICAM-1), IL-6, GM-CSF, IL-8, CXCL-1] upon treatment has been associated with the activation of stress-associated mitogen-activated protein kinase (MAPK) signaling pathway [Jun N-terminal kinase (JNK) and P38 MAPK] (Toste et al., 2016). Finally, the indirect effect of CT on CAFs secretome may also occur due to the dysregulated crosstalk between cancer cells and CAFs. For instance, Nab-paclitaxel treatment increases CXCL-10 expression in pancreatic cancer cells, leading to lower IL-6 secretion by CAFs subsequently impairing migration and invasive capabilities of cancer cells (Feng et al., 2018).

#### Radiotherapy

Similarly to CT, radiotherapy (RT) has been reported to impact the secretome of CAFs (Figure 3). Rødningen and colleagues showed that ionizing radiation altered the fibroblasts expression of genes involved in ECM remodeling as well as Wnt and IGF signaling to support cancer stemness and resistance to therapy (Rødningen et al., 2005; Wang Z. et al., 2019). Persistent DNA damage, induction of senescence, and TGF-beta pathway activation rather than cellular death are some of the proposed mechanisms modulating CAFs secretome upon RT (Ansems and Span, 2020). Importantly, a vast body of evidence suggests that RT enhances paracrine signaling between fibroblasts and cancer cells through the IGF and TGF-beta pathways (Barker et al., 2015; Tommelein et al., 2018). Promotion of EMT, enhanced cancer stemness properties, and increased resistance to therapy are among the various effects exerted by RT-activated CAFs (Arshad et al., 2015; Barker et al., 2015). For instance, IGF-1 activates the IGF-1R/AKT/mTOR survival pathway in CRC, and CXCL-12 promotes EMT and invasion in pancreatic cancer (Li D. et al., 2016; Tommelein et al., 2018). Alternatively, RT-activated CAFs may also support intra-tumoral angiogenesis (Hellevik et al., 2013). Interestingly, several studies have reported that the immunoregulation exerted by CAFs remains unaltered after ionizing RT (Hellevik et al., 2013; Gorchs et al., 2015; Berzaghi et al., 2019). Thus, combination with immune-targeted therapies may be an attractive clinical approach for RT-treated patients.

#### Targeted Therapy

Originally designed to be specific to cancer cells, targeted therapies may also produce a range of undesirable off-target effects on stromal cells and particularly on CAFs secretome. For instance, specific secretion of HGF and neuregulin (NRG)-1 by fibroblasts upon exposure to vemurafenib -a BRAF inhibitor-was reported in melanoma (Fedorenko et al., 2015). Of note, the activation of MET by HGF has been suggested as a potential mechanism of acquired tyrosine kinase inhibitor (TKI) resistance to gefitinib in epidermal growth factor receptor (EGFR)-mutant non-small cell lung cancer (NSCLC) (Yano et al., 2008) and to vemurafenib itself in BRAF-mutant melanoma (Wilson et al., 2012). In PCa, HGF and NRG-1 have been identified as non-cell-autonomous drivers of antiandrogen resistance. In this setting, NRG-1/epidermal growth factor receptor 3 (HER-3) signaling axis blockade demonstrated promising tumor growth suppression (Zhang Z. et al., 2020). More recently, Hirata and colleagues reported alternative CAF-induced resistance to BRAF inhibition. In this setting, BRAF inhibitor reprograms CAFs by enhancing platelet-derived growth factor receptor (PDGFR) activity, thus increasing ECM production –thrombospondin (THBS)-1/2, tenascin C (TNC), or POSTN, among other matrix components– and stiffness to potentiate cancer cells tolerance to treatment (Hirata et al., 2015). These data illustrate how CAFs functions can be affected even by targeted therapies to enable the emergence of resistance to treatment.

### Epigenetic Regulation of CAFs Secretome

CAF phenotype may be driven by epigenetic deregulation rather than by somatic mutations (Qiu et al., 2008; Bianchi-Frias et al., 2016; Pidsley et al., 2018). DNA methylation, histone modifications, chromatin remodeling, and non-coding RNA–micro RNA (miRNA) and long non-coding RNA (lncRNA)–are the well-described molecular mechanisms behind the epigenetic CAF reprogramming and have been thoroughly reviewed elsewhere (Marks et al., 2016; Melissari et al., 2020; Pan and Zheng, 2020). Interestingly, several studies support the hypothesis that an *epigenetic switch* would initiate the activation process leading to a stable CAF cell state with tumor-supportive secretory phenotype (Albrengues et al., 2015; Kalluri, 2016).

For instance, multiple genes coding for secreted factors, such as IL-1a, CCL-5, and CCL-26; show differential hypomethylation patterns in CAFs and are consequently overexpressed in pancreatic cancer. In this case, paracrine lactate secreted by PDAC cells leads to ten-eleven translocation (TET) enzyme activation triggering the epigenomic reprogramming (Bhagat et al., 2019). Remarkably, different epigenetic mechanisms can merge into the same signaling cascade as happening for the STAT-3 pathway. Indeed, SOCS-1 methylation and downregulation in PDAC-associated CAFs enhance STAT-3-induced IGF-1 expression (Xiao et al., 2016) to promote the survival and proliferation of pancreatic cancer cells (Kopantzev et al., 2017). Alternatively, LIF-induced methylation of Shp-1 promoter abrogates its expression leading to a STAT-3-mediated CAF pro-invasive phenotype (Albrengues et al., 2015).

In conclusion, in the absence of genomic mutations, epigenetic alterations may be seen as key determinants of CAF phenotype and could become promising targets for cancer treatment. Importantly, several clinical trials are currently exploring the benefits of DNA methylation therapies in solid tumors including colorectal (NCT01896856), pancreatic (NCT03264404), or prostate cancer (NCT03572387). Even though cancer cells are the intended target, the above-mentioned data suggest a potential effect of DNA methylation therapies over CAFs that will need to be carefully assessed in treated patients.

## CAFS Secretome as Determinant of Tumor Development

### Cancer Cells Self-Renewal

**Figure 4:**
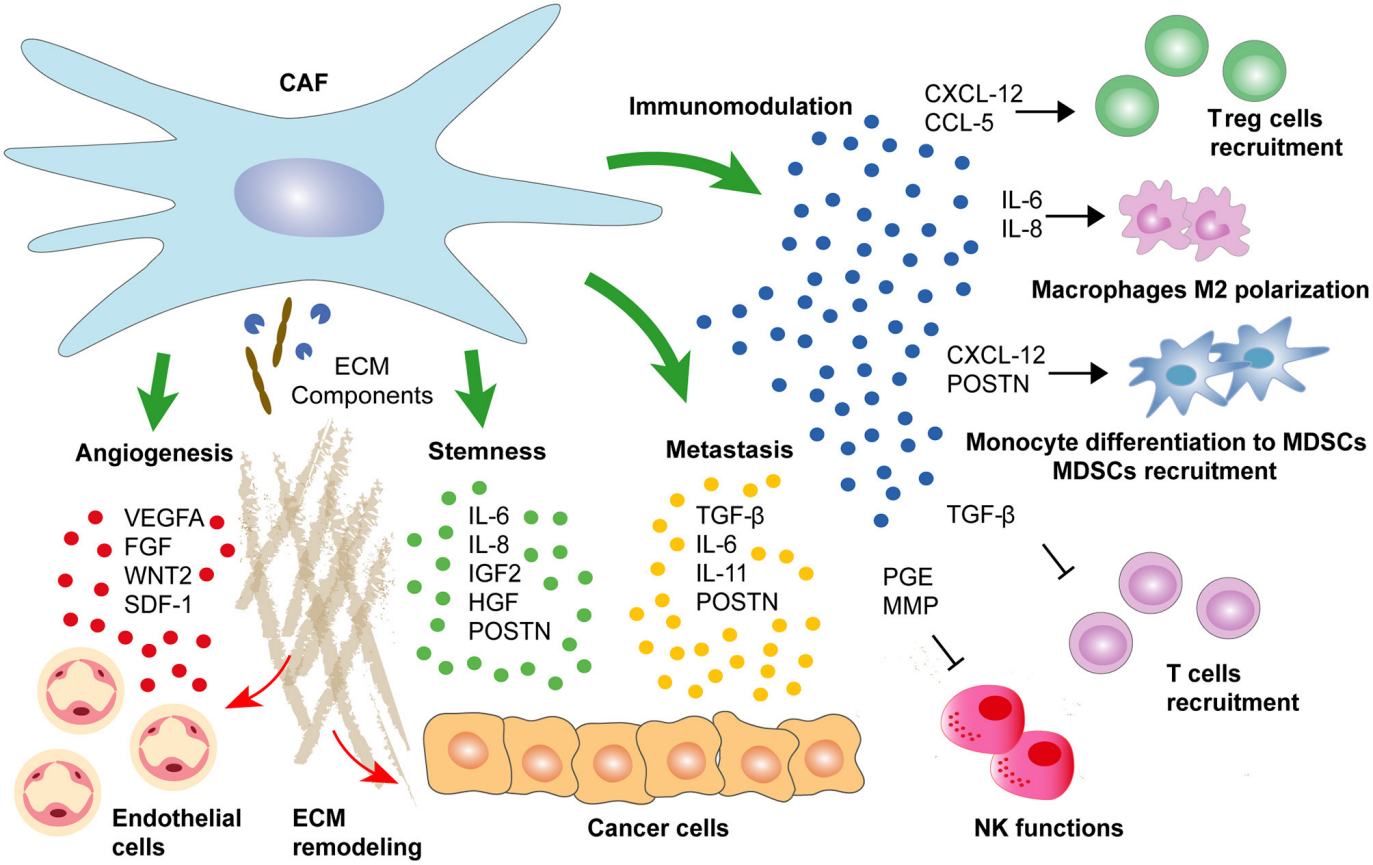
CAFs secretome determines cancer progression. CAF-secreted factors regulate cancer development by enhancing directly epithelial cancer cells self-renewal and aggressiveness. Alternatively, a range of CAF-secreted factors may support progression to metastasis by maintaining an immunosuppressive environment or by enhancing pro-tumorigenic angiogenesis.

A vast body of evidence indicates that CAFs secretome may contribute to tumor progression by enhancing cancer stemness (Figure 4). Indeed, CAF-secreted IGF-2 as well as IL-6 and IL-8 produced by CD-10+ GPR-77+ CAFs are promoting cancer stemness and tumor formation in lung cancer (Chen et al., 2014; Su et al., 2018). Similarly, PCa-derived CAFs with decreased expression of AR promote stemness in cancer cells through IFN-γ and M-CSF secretion (Liao et al., 2017). Importantly, cancer stemness induced by CAF-secreted factors may involve different pathways. For instance, PI3K/AKT pathway activation drives the progression of anal squamous cell carcinoma through IGF-2 secretion by the PDGFRB+ CAFs population (Cacheux et al., 2019). Similarly, IGF-2-secreting CAFs promote tumor regrowth and decreased latency after primary resection in CRC (Unger et al., 2017). Alternatively, NOTCH signaling may be triggered by CAF-derived CCL-2 to induce stem cell features as observed in BC cells (Tsuyada et al., 2012). Finally, WNT signaling activation by CAF-secreted HGF in colorectal adenocarcinomas or by CAF-secreted POSTN in head and neck squamous cell carcinoma and BC cells may also promote cancer stemness and further metastatic initiation (Vermeulen et al., 2010; Malanchi et al., 2011; Yu et al., 2018).

In addition to cytokine-driven signaling, cancer cells self-renewal and aggressiveness may also depend on metabolic cues originating from the TME (reviewed in Reina-Campos et al., 2017; Li et al., 2020). Specifically, CAFs originating lactate and glutamine production as well as autophagic alanine secretion are sustaining cancer cells metabolism, stemness, and progression to metastasis (Fiaschi et al., 2012; Curry et al., 2013; Sousa et al., 2016; Yang L. et al., 2016). The specific mechanisms enabling CAFs to deliver distinct nutrients to cancer cells are being explored. However, CAFs response to the surrounding constraints, such as the epigenetic reprogramming induced by chronic hypoxia, is likely to play a relevant role in metabolic reprogramming of CAFs (Becker et al., 2020).

### Metastatic Cancer Cells Spreading

As mentioned above, CAF-secreted factors may also drive cancer progression by enhancing the metastatic potential of cancer cells (Figure 4). For instance, while TGF-beta activates different genetic programs in a wide range of stroma cells, the main contributor to its pro-tumorigenic influence seems to be an autocrine loop maintaining the secretion of pro-metastatic factors by CAFs (Calon et al., 2012). In addition, CAF-secreted TGF-beta promotes paracrine reprogramming of epithelial cancer cells by inducing EMT in tumors of distinct origin (Zhuang et al., 2015). In CRC, TGF-beta induces IL-13 expression in fibroblasts, and subsequent TGF-beta/IL-13 synergy activates an EMT program in epithelial cells (Scharl et al., 2013). Alternatively, Calon and colleagues described TGF-beta-activated CAFs secreting IL-6 and IL-11 leading to enhanced STAT-3-dependent survival and spreading of metastatic CRC cells (Calon et al., 2012). Similar observations were made in gastric cancer where CAF-secreted IL-6 induced EMT and metastasis through STAT-3 pathway activation (Wu et al., 2017). Alternatively, CAF-secreted vascular cell adhesion protein (VCAM)-1 has been shown to stimulate lung cancer cells migration and invasion (Zhou et al., 2020).

Besides their autocrine and paracrine activities regulating progression to metastasis, CAFs-secreted factors may also support cancer spreading by influencing ECM deposition at both the primary and metastatic sites. For example, Chakravarthy and colleagues described that TGF-beta-activated CAFs could induce the expression of large ECM components related to cancer progression (Chakravarthy et al., 2018). In this regard, TGF-beta-activated CAFs may secrete POSTN in order to increase proliferation, motility, and invasive properties in head and neck cancer (HNC) cells (Qin et al., 2016). Similarly, CAF-secreted POSTN may also promote tumor progression in CRC through YAP/TAZ activation in cancer cells (Ma et al., 2020). Of note, Deng and colleagues showed that the YAP-activated Wnt/β-catenin pathway promotes colon tumorigenesis (Deng et al., 2018). Hence, the overall data suggest a complex paracrine regulation of the Wnt pathway in cancer cells by TGF-beta-activated CAFs through POSTN secretion and YAP activation in order to enhance tumor progression and metastasis formation.

### Immunomodulation

Somewhat contradictory evidences of CAFs immunoregulatory functions have been described during cancer progression (Figure 4). While increased abundance of CAFs clearly predicts immune exclusion and immunotherapy failure, a complete depletion of CAFs was associated with decreased intra-tumoral immune infiltration (Özdemir et al., 2014; Chakravarthy et al., 2018). One reason explaining opposite functions of CAFs in modulating immune infiltration may reside in the fact that different CAFs subpopulations with distinct properties are coexisting inside the tumor. For example, among the four CAF subsets identified in BC, only CAF-S1 subtype characterized by high FAP expression promoted immunosuppression by secreting CXCL-12 and enhancing immunosuppressive T regulatory cells (T_reg_) capacity to block T effector cells proliferation (Costa et al., 2018). Importantly, FAP+ CAFs are also the main producers of CXCL-12 in PDAC, and their specific depletion increases immunological control over tumor growth (Kraman et al., 2010; Feig et al., 2013). In this setting, the decreased expression of CXCL-12 leads to intra-tumoral T cell accumulation, which in turn synergizes with immuno- or chemotherapy to reduce cancer cells abundance (Feig et al., 2013; Sherman et al., 2014).

In addition to CXCL-12, a wide range of CAF-produced chemokines has been associated with tumor immunosuppression. For example, CAF-secreted CCL-5 induces CD4+CD25+ T_reg_ cells in BC (Tan et al., 2011; Costa et al., 2018). Notably, CCL-5 dependent T_reg_ cells recruitment was associated with lung metastasis in BC mouse model (Tan et al., 2011). CAFs also participate in immunosuppressive myeloid cell recruitment and differentiation by secreting SDF-1 to attract monocytes and to induce their differentiation into myeloid-derived suppressor cells (MDSCs) contributing to cancer immune evasion (Gabrilovich and Nagaraj, 2009; Deng et al., 2017). Alternatively, Wang and colleagues showed that CAF-secreted POSTN promoted MDSCs infiltration to establish a pre-metastatic niche during breast tumor metastasis through AKT and STAT-3 pathway activation (Wang Z. et al., 2016). Similarly, FAP+ CAF-secreted CCL-2 mediates STAT-3 pathway activation in MDSC to promote liver tumor growth and to suppress T cells proliferation during colorectal carcinogenesis (Chun et al., 2015; Yang et al., 2016). Alternatively, CAF-secreted IL-6 and IL-8 may promote monocytes differentiation into M2 macrophages and further recruitment in the tumor to diminish natural killer (NK) cells cytotoxicity against CRC cells (Cho et al., 2018; Zhang et al., 2019). Finally, CAFs may directly interfere with NK anti-tumoral functions through prostaglandin E2 (PGE2) secretion or MMPs production decreasing NK receptor ligands expression in tumor cells, thus reducing NK receptor-dependent cytotoxic activity (Balsamo et al., 2009; Ziani et al., 2017).

As mentioned earlier, CAFs are major producers of TGF-beta, a key contributor to tumor immunosuppression (Calon et al., 2015; Batlle and Massagué, 2019). For instance, TGF-beta produced by CAFs was correlated with CD8+ T cells exclusion, which in turn has been associated with resistance to anti-PD-L1 antibody-based immune checkpoint inhibitor therapy (Mariathasan et al., 2018). In this context, TGF-beta pathway blockade through TGF-beta receptor (TGFBR)-1 inhibition or TGF-beta blocking antibody enabled T cells infiltration into the tumor, restoring the anti-tumor immunity induced by the anti-PD-L1 antibody in BC and CRC pre-clinical models (Mariathasan et al., 2018; Tauriello et al., 2018). In contrast, TGFBR-2 deletion in BC induces MDSCs recruitment to the TME, reducing T cell proliferation and activation (Yang et al., 2008). Interestingly, TGFBR-2 deletion has been associated with the increased expression of stromal TGF-beta leading to MDSCs recruitment of indifferent cancers including HNC or PCa (Lu et al., 2006; Franco et al., 2011). These data suggest that the genetic ablation of TGFBR-2 enhances the secondary activation of TGF-beta that may indirectly promote intra-tumoral immunosuppression.

### Angiogenesis

Neovascularization is an important process during tumorigenesis. Interestingly, CAFs are the main source of VEGF-A, the most potent pro-angiogenic factor, which binds to its cognate VEGF receptor (VEGFR)-2 expressed on endothelial cells (Figure 4) (Fukumura et al., 1998; Apte et al., 2019). Noma and colleagues showed that TGF-beta pathway activation in CAFs was essential to induce VEGF secretion (Noma et al., 2008). In addition, Sánchez-Elsner and colleagues demonstrated a synergistic cooperation between SMADs and hypoxia-inducible factor (HIF)-1 alpha proteins to drive VEGF expression, suggesting a potential link between TGF-beta pathway and hypoxia to promote tumor angiogenesis (Sánchez-Elsner et al., 2001). Nevertheless, there is controversial evidence about the role of TGF-beta in tumor angiogenesis suggesting a differential expression of TGF-beta ligands during different phases of angiogenesis (Pardali et al., 2010). For instance, Liu and colleagues showed that TGFBR-1 inhibitor and VEGF synergistically induced tumor angiogenesis through α5-integrin upregulation (Liu et al., 2009). In contrast, TGF-beta-TGFBR-1 signaling has been involved in the promotion of BC angiogenesis through MMP-9 upregulation and through CAF-secreted TGF-beta and SDF-1 in HCCs (Safina et al., 2007; Yang J. et al., 2016).

In addition to promoting VEGF-dependent angiogenesis, CAFs may enhance tumor angiogenesis through additional paracrine signaling between stromal and cancer cells (Orimo et al., 2005; Unterleuthner et al., 2020). For instance, CAF-derived CXCL-12 leads to CXCL-8 secretion by PDAC cells inducing new vessel formation by endothelial cells (Matsuo et al., 2009). Alternatively, CAF-secreted galectin-1 induces VEGF-A production by BC cells to promote tumor angiogenesis (Tang et al., 2016). FGF has also been recognized as a potent pro-angiogenic factor involved in tumor angiogenesis (Compagni et al., 2000). Correspondingly, Pietras and colleagues demonstrated that cervical carcinoma-derived CAFs are secreting FGF-7 and FGF-2, inducing both epithelial proliferation and tumor angiogenesis (Pietras et al., 2008).

Finally, CAFs not only are directly promoting tumor angiogenesis by secreting pro-angiogenic factors but also act indirectly by participating in ECM remodeling (Figure 4) (De Palma et al., 2017). For example, CAFs may support vascularization through biomechanical activity and ECM stiffness promoted by MMP activity (Bordeleau et al., 2017; Sewell-Loftin et al., 2017). Importantly, CAFs are the most important suppliers of ECM-associated proteins essential to vascular formation including TNC production resulting in pro-angiogenic paracrine signaling (Newman et al., 2011; Rupp et al., 2016).

### Resistance to Anti-cancer Therapy

In addition to modulating tumor progression, CAFs secretome may also promote resistance to systemic and targeted therapies. For instance, CAF-secreted SDF-1 upregulates the expression of special AT-rich sequence-binding protein-1 (SATB-1) in pancreatic cancer cells, which not only sustains pancreatic tumor growth but also mediates gemcitabine resistance (Wei et al., 2018). Furthermore, CAF-derived IGF-1 and IGF-2 induce CT resistance in pancreatic cancer (Ireland et al., 2016). IGF-2 also increases insulin receptor/IGF 1 receptor (IGF1R) axis activation in cancer cells to enhance resistance to anti-EGFR-targeted therapy in cholangiocarcinoma (Vaquero et al., 2018). In the same line, fibroblast-secreted HGF activates both the MAPK and PI3K/AKT pathways contributing to BRAF-targeted therapies' primary resistance in melanoma and in a subset of colorectal and glioblastoma cancer cells (Straussman et al., 2012).

Regarding hormonotherapy, CAFs-secreted factors contribute to estrogen receptor alpha (ER-α) phosphorylation in BC cells, thereby promoting tamoxifen resistance (Pontiggia et al., 2012). In addition, Li and colleagues reported that letrozole, an aromatase inhibitor lowering estrogen production, had opposite functional effects on CAFs secretome (Li K. et al., 2016). Letrozole reduces CCL-2, CCL-5, and CXCL-1 expression in CAFs, possibly contributing to its efficacy against BC cells. Conversely, letrozole also increases CAFs secretion of POSTN, a factor involved in BC progression and metastasis (Kyutoku et al., 2011; Li K. et al., 2016). In PCa, Zhang and colleagues, described that CAF-secreted NRG-1 activates the HER-3 signaling pathway leading to resistance to antiandrogen therapy (Zhang Z. et al., 2020). Notably, NRG-1 expression in PCa-derived CAFs is increased after antiandrogen therapy that may contribute to potentiate resistance to treatment (Zhang Z. et al., 2020).

Consequently, the presence in the tumor of a range of secreted factors involved in resistance to therapy may be the result of CAFs' own response to anti-cancer treatment. Indeed, CT induces senescence-associated secretory phenotype in fibroblasts, typically characterized by the increased secretion of cytokines, chemokines, and growth factors previously associated with tumor progression and treatment resistance (Demaria et al., 2017). More specifically, Sun and colleagues demonstrated that primary prostate fibroblasts increased WNT-16B expression in response to CT. In this setting, WNT-16B enhanced prostate tumor growth and diminished CT cytotoxicity against cancer cells (Sun et al., 2012). Finally, previously mentioned IL-11 secreted by CAFs upon cisplatin-based CT induced STAT-3 pathway activation and chemoresistance in lung cancer cells (Tao et al., 2016).

## Prognostic and Predictive Value of CAFS Secretome

The identification of tumor features predicting prognosis, risk of relapse, and benefit from treatment is absolutely essential for clinical decision-making in oncology. In this sense, a better understanding of the TME has provided multiple prognostic and diagnostic biomarkers in cancers of distinct origin (Bremnes et al., 2011). For instance, CAFs biomarkers (Ha et al., 2014; Dourado et al., 2018), tumor:stroma ratio quantification (Kemi et al., 2018; Vangangelt et al., 2018), and stromal gene expression profiling (Finak et al., 2008; Frings et al., 2013; Calon et al., 2015) are powerful tools predicting clinical outcome. However, addressing the exclusive impact of CAFs secretome on cancer prognosis remains a major challenge, and to our knowledge, there is no example of CAF-secreted biomarkers currently applied to the clinical setting.

Cytokines and chemokines have been proposed as prognostic factors in different types of cancer. Yet, their pleiotropic nature and multiple cellular origin as well as their distinct releasing patterns make their use as biomarkers especially challenging. Circulating IL-6 has been associated with cancer progression and poor prognosis in melanoma, BC, and gastrointestinal tumors, among others (Salgado et al., 2003; Hoejberg et al., 2012; Vainer et al., 2018), but only few studies have assessed IL-6 cellular origin. In bladder cancer patients for instance, CAF-derived IL-6 and ACTA-2 (αSMA coding gene) co-upregulation correlated with poor survival (Goulet et al., 2019). In esophageal adenocarcinoma, although IL-6 serum levels did not correlate with patients' outcome, the expression of ADAM-12, a surrogate marker for IL-6-producing CAFs, predicted poor prognosis after neoadjuvant chemoradiation (Ebbing et al., 2019). CXCL chemokines are additional examples of promising prognosis biomarkers associated with CAFs functions. Indeed, CAF-derived CXCL-14 expression was correlated with shorter recurrence-free survival in estrogen receptor negative, triple negative, and basal-like BC (Sjöberg et al., 2016), as well as in additional tumor types (reviewed in Westrich et al., 2020). Similarly, CXCL-1 and CXCL-8 positivity in CAFs was significantly associated with poor prognosis in gastric cancer patients (Naito et al., 2019), whereas CXCL-8,−10, and−11 CAFs expression correlated with resistance to neoadjuvant CT and poor prognosis in BC (Xu et al., 2020).

As previously mentioned, CAFs-secreted growth factors, such as HGF or IGFs, are involved in resistance to targeted therapy and may be of use to predict response to treatment. Indeed, HGF expression was correlated with innate resistance to BRAF inhibition, and increased HGF plasma levels predicted worse overall survival (OS) and progression-free survival (PFS) in BRAF-mutant melanoma patients (Straussman et al., 2012; Wilson et al., 2012). Alternatively, CAF-secreted IGF-binding proteins have been proposed as a potential therapeutic target and prognostic biomarker in pancreatic cancer and other tumor types (Thomas and Radhakrishnan, 2020).

Additional CAFs-secreted proteins, such as VCAM-1, THBS-2, and POSTN, involved in cancer progression were recently evaluated as potential actionable biomarkers. In lung cancer, the soluble fraction of VCAM-1 predicted relapse and lower OS, whereas high POSTN expression was associated with shorter PFS in ovarian cancer patients (Ryner et al., 2015; Zhou et al., 2020). Of note, a recent meta-analysis confirmed the value of POSTN as a biomarker predicting poor outcome in different solid tumors (Yang et al., 2020). Finally, increased THBS-2 predicted decreased OS in CRC patients (Wang X. et al., 2016).

CAF-derived exosomes transferring miRNA (Au Yeung et al., 2016; Fang et al., 2019; Qin et al., 2019) and lncRNA (Qu et al., 2016) to tumor cells have been reported to promote treatment resistance in different tumor types and were also evaluated as potential predictive biomarkers. In this context, serum detection of plasma exosomal miR-196a levels correlated with tumor size, lymph node metastasis, advanced tumor stage, and poor OS while accurately discriminating chemoresistant and sensitive patients in HNC (Qin et al., 2019). Of note, suppressing or interfering with CAF-derived exosomes transference to cancer cells has been proposed as a novel therapeutic approach (Li et al., 2017).

Similarly to soluble factors, many ECM-related products are contributing to the predictive power of stromal-originating gene signatures (Andriani et al., 2018; Yuzhalin et al., 2018; Jiang et al., 2019). ECM remodeling enzymes, such as MMPs and tissue inhibitors of MMP (TIMP), which are not exclusively but mainly released by CAFs, have also been suggested to predict cancer progression and response to treatment (Liu et al., 2019). Among them, Eiró et al. assessed the potential influence over patients' prognosis of TIMP-2 expression by CAFs at the tumor center and the invasive front of early stage BC. TIMP-2 resulted to be a potent poor outcome predictor at both locations (Eiró et al., 2015). Many members of the MMP family promoting tumor invasion through ECM degradation are also impacting cancer prognosis. For instance, increased MMP-1 expression by CAFs was associated with high risk of relapse in stage II CRC, and CAFs expression of MMP-9,−11, and−13 correlated with shorter relapse-free survival in BC (González et al., 2009; Eiro et al., 2019). Remarkably, the relevance of MMPs for prognosis might be cancer specific, as illustrated by MMP-2, which predicts poor OS and PFS in NSCLC but associates with better survival in patients with high grade BC (Leinonen et al., 2008; Niemiec et al., 2013).

Along this line, other CAF-secreted ECM components, such as collagens, have been recently recognized as important contributors to cancer progression and as potential liquid biopsy biomarkers in different tumor types (Giussani et al., 2019). Serum level of PRO-C3 –a biomarker of collagen III production– is a promising example predicting poor OS in PDAC patients and may be a non-invasive actionable biomarker for desmoplasia-targeting therapies (Willumsen et al., 2019). Interestingly and probably due to TGF-beta capacity to stimulate collagens production by CAFs, serum levels of collagen fragments have been associated with response to TGF-beta-targeted therapy (Nissen et al., 2019).

Overall, these exciting findings are calling for further validation of CAF-derived biomarkers in order to improve the standard of care and decision-making in oncology. However, it is worth noting that methodological variability will be especially relevant when transferring biomarkers from bench to bedside. Indeed, evaluating either a single or a panel of factors through distinct techniques may complicate the validation of CAFs secretome-based prognostic and predictive tools in the clinical setting (Paulsson and Micke, 2014). Spatial and temporal heterogeneity (see the Determinants of CAFs Secretome section) may be an additional issue that could be overcome by liquid biopsy, a non-invasive method allowing real-time evaluation of CAF-secreted biomarkers circulating in the bloodstream (Herrera et al., 2019).

## CAF-Secreted Factors as a Therapeutic Target

### Targeting the CAFs Secretome Regulating Cancer Cells Proliferation

Since CAFs complete depletion or blockade of fibroblast-rich tumor stroma formation resulted in decreased anti-tumor immune infiltration and more aggressive tumors, recent strategies have rather focused on the regulation of CAFs originating paracrine and autocrine signaling (Özdemir et al., 2014; Rhim et al., 2014). For instance, CAFs reprogramming by vitamin A and vitamin D was shown to inhibit tumor-supportive secretome associated with cancer progression (Froeling et al., 2011; Sherman et al., 2014). Remarkably, gemcitabine and either vitamin A or vitamin D regimen resulted in significant tumor burden reduction in PDAC pre-clinical models (Sherman et al., 2014; Carapuça et al., 2016). Hence, a phase II trial is currently evaluating the combination of CT and vitamin D in PDAC patients (NCT03415854). Yet, a previous phase II trial did not show any survival benefit with high doses of vitamin D3 compared with standard doses in combination with CT in metastatic CRC patients (Ng et al., 2019).

Importantly, vitamin A and vitamin D reprograming strategies are both associated with TGF-beta –a key autocrine and paracrine mediator of CAFs signaling– pathway inhibition. Indeed, vitamin D receptor ligands decrease fibroblast activation by TGF-beta (Ding et al., 2013), whereas all-trans retinoic acid (ATRA) –the active metabolite of vitamin A– inhibits the fibroblasts capacity to release active TGF-beta, thus impeding autocrine TGF-beta activation (Sarper et al., 2016). Of note, ATRA reprogramed fibroblasts are in addition reducing Wnt–β-catenin signaling in the surrounding cancer cells through SFRP-4 secretion (Froeling et al., 2011).

TGF-beta has emerged as a potential therapeutic target in oncology. Notably, TGF-beta inhibitor in combination with gemcitabine improved the OS in locally advanced and metastatic PDAC patients (Melisi et al., 2018). A phase I/II trial is currently testing the ability of TGF-beta inhibitor to restore the sensitivity to CT in patients with TGF-beta-activated program in metastatic CRC resistant to CT (NCT03470350). The analysis of the TGF-beta-activated program in CAFs provided several additional therapeutic targets. Among them, IL-6 and IL-11 are secreted interleukins activating STAT-3-dependent survival and spreading of metastatic CRC cancer cells (Calon et al., 2012). IL-11 or IL-6 inhibitors displayed potent anti-cancer activity in pre-clinical models of cancer of distinct origins (Coward et al., 2011; Putoczki et al., 2013; Song et al., 2014). Siltuximab, an IL-6 inhibitor, did not show benefit as monotherapy in CT-pretreated castration-resistance PCa patients (Dorff et al., 2010). However, additional IL-6 pathway inhibitors are currently tested in combination with CT in patients with breast (NCT03135171), pancreatic (NCT04258150, NCT02767557), or liver cancer (NCT04338685). Among CAF-secreted molecules, ECM components have also raised special interest as therapeutic targets. Yet, clinical trials failed to demonstrate the clinical benefit from COL-3, MMP-2, and MMP-9 inhibitors in patients (Chu et al., 2007). Similarly, PEGPH20, a PEGylated recombinant human hyaluronidase, did not add benefit to standard CT in PDAC patients (Tempero et al., 2020).

Off-target effects and indirect resistance to treatment involving CAFs functions may explain the lack of benefit of current stroma-targeting therapies. For example, Casanovas and colleagues described that VEGFR blockade induces tumor hypoxia and paradoxically triggers pro-angiogenic factors production, such as FGF family members (Casanovas et al., 2005). Likewise, CAFs derived from anti-VEGF-resistant tumors are secreting PDGF-C in order to reactivate tumor angiogenesis (Crawford et al., 2009). A potential solution may come from multi-targeted therapies, such as nintedanib, a triple angiokinase inhibitor blocking VEGFR, FGFR, and PDGFR. Nintedanib reduces the CAFs expression of IL-6, IL-8, VEGF, and VCAM-1 as well as OPN and showed important clinical benefit in combination with CT in NSCLC patients (Hilberg et al., 2008; Reck et al., 2014; Yamanaka et al., 2020).

### Overcoming CAFs-Induced Immunosuppression

Several preclinical studies have recently underscored the relevance of TGFBR-1 targeting in order to activate an anti-tumor immune response. TGFBR-1 inhibitor (galunisertib) and anti-PD-L1 combination treatment increased intra-tumoral T cell infiltration and activation, thereby promoting a potent anti-tumor response in CRC and BC pre-clinical models. Characteristically, this effect was associated with a reduction of fibroblasts activation and an increased anti-tumor immune genes expression (Holmgaard et al., 2018; Mariathasan et al., 2018; Tauriello et al., 2018). Curiously, anti-PD-L1 treatment alone enhanced the expression of TGF-beta activated CAFs biomarkers, suggesting that TGF-beta pathway inhibition is essential to enhance anti-PD-L1 therapy (Holmgaard et al., 2018). In this context, two phase I trials are being conducted to evaluate the benefit of galunisertib in combination with immune checkpoint inhibitors (anti-PD-1, anti-PD-L1) in refractory metastatic cancer patients (NCT02423343, NCT02734160). In contrast, Zhao and colleagues showed that another TGFBR-1 inhibitor (TEW-7197) failed to increase anti-PD-L1 treatment efficacy in melanoma mouse model. TEW-7197 promoted fibroblasts proliferation and diminished PD-L1 expression in cancer cells through MMP-9 secretion by CAFs. Interestingly, the authors reported increased TGFBR-2 expression upon treatment (Zhao et al., 2018). In this context, previously mentioned TGFBR-2-induced secondary activation of TGF-beta could potentially contribute to CAFs activation and to combination treatment failure (Lu et al., 2006; Franco et al., 2011).

Bintrafusp alfa, a bi-functional fusion protein composed of an anti-PD-L1 antibody and a TGF-beta “trap,” was recently designed to potentiate immune checkpoint and TGF-beta inhibitors regimen. Bintrafusp alfa induces tumor regression and decreased spontaneous metastasis in a CRC mouse model by activating CD8+ T cells and NK cells while abrogating CAFs activation in the tumor (Lan et al., 2018). Clinical benefit of bintrafusp alfa is currently under evaluation in several cancer patients' trials including NSCLC (NCT03840902), BC (NCT03579472), HNSCC (NCT04247282), and CRC (NCT03436563).

Remarkably, previously mentioned ATRA-mediated CAFs reprograming increases T cells infiltration in PDAC while blocking CAFs autocrine TGF-beta activation (Ene-Obong et al., 2013; Sarper et al., 2016). In view of the above-mentioned data on TGF-beta inhibition, pancreatic cancer patients who will be treated with ATRA (NCT04241276) may as well benefit from combination regimen with immune checkpoint inhibitors.

Finally, CAF-secreted factors, such as FN and TNC, have been targeted for antibody-based delivery of immune enhancers specifically to the tumor site in order to concentrate their effect on cancer tissue and decrease treatment-associated toxicity on healthy tissue (Pasche and Neri, 2012). For example, L19-TNF and L19-IL-2 immunocytokines conjugate an antibody-recognizing FN with cytokines (TNF-α and IL-2, respectively) (Neri, 2019). Preclinical data already suggested the robust anti-cancer potential of L19-based immunocytokines (Lieverse et al., 2020), and phase II and III trials are currently testing the clinical benefit of L19-TNF-a and L19-IL-2 in patients with soft tissue sarcoma (NCT03420014) or melanoma (NCT02938299, NCT03567889).

## Conclusions

From a general perspective, CAFs should be considered as a heterogeneous and dynamic stromal component which evolution to distinct functional subpopulations is paralleling the tumorigenic process. Spatio-temporally regulated factors and crosstalk with other TME components drive this co-evolution from local tissue resident fibroblasts and other cell types to distinct CAFs subtypes. Indeed, the intra-tumoral location effect over CAFs function is determined by spatial distribution of tumor cell-derived factors (Figure 1). Among them, autocrine or paracrine communications and EVs interchange have been described to define CAF phenotypes. However, the influence of other cell populations–infiltrating immune cells, endothelial cells–, and biomechanical stress (ECM stiffness) also differentially distributed within the TME may play an important role in modulating CAFs behavior. Among other potential functions associated with CAFs, there is solid evidence of several well-defined cell sub-specializations with either contractile and ECM remodeling functions or secretory and immunomodulating functions (Figure 2).

The potential role of CAFs secretome for predicting patient's outcome and response to treatment has also been intensively investigated. Overall findings illustrate the potential benefit of using CAFs secretome biomarkers to improve patient's selection and treatment follow-up. Notwithstanding their potential as actionable biomarkers, current knowledge is still mostly providing descriptive information of individual secreted factors but does not advocate for their translation into the clinical setting. Comprehensive -omics analyses are currently being used for an extensive characterization of CAFs secretome in order to discover and validate robust biomarkers and novel targets within the stroma (Principe et al., 2018). In this sense, liquid biopsy appears to be a promising method for real-time evaluation of the different components of CAFs secretome, allowing the detection of CAF-derived soluble factors, CAF-derived exosomes, and even circulating CAFs as potential biomarkers (Herrera et al., 2019).

Recent findings suggest a clear impact of both systemic and targeted anti-cancer therapies upon CAFs secretome that will need to be carefully assessed in view of patients' response to treatment (Figure 3). Understanding the impact of CAFs secretome on treatment resistance after therapy exposure will provide original tools to monitor patient's response. The implementation of clinical criteria evaluating components of the CAFs secretome will help in refining patients' selection for suitable therapies and improve oncological outcomes.

While CAFs contribution over tumor development is still a matter of research, CAFs secretome has already demonstrated its potential as a target for original therapeutic strategies in a wide range of cancers (Figure 4). However, a better understanding of CAFs secretome has also underscored the importance of defining the cell-type specific response to secreted factors. For instance, TGF-beta is a pro-metastatic cytokine currently targeted in multiple clinical trials (NCT04031872, NCT02452008, NCT03834662, NCT04574583). Yet, TGF-beta is also considered as a tumor suppressor due to its cytostatic effect on cancer cells (Akhurst and Hata, 2012). Another example is IL-11, a pro-metastatic factor that can be successfully targeted by IL-11 signaling antagonist to reduce cancer progression (Putoczki et al., 2013). Until recently, IL-11 was better known for its capacity to stimulate platelet production, and for decades, cancer patients have been treated with rhIL-11 to overcome CT-induced thrombocytopenia (Isaacs et al., 1997; Cairo et al., 2005; Wu et al., 2012). These findings underscore the importance of carefully assessing every cell-type specific response to secreted factors before therapeutic translation into the clinical setting.

CAFs may not be understood and targeted as a unique family anymore, and a detailed definition of CAFs secretome is still an unmet need. For instance, CAFs and senescent fibroblasts coexisting within the TME are able to secrete equivalent factors. Hence, CAFs secretome and senescence-associated secretory phenotype (SASP) may have overlapping tumor-promoting effects (Sahai et al., 2020).

Different treatment strategies, such as targeting a specific functional subtype of CAFs, reprogramming CAFs backwards to a tumor-suppressor phenotype, or even switching between distinct functional subtypes, are currently being addressed. Along this line, a major challenge in precisely defining CAFs heterogeneity and therapeutically targeting CAFs secretome will reside in a better comprehension of CAFs spatiotemporal evolution during tumor progression.

Overall, it is reasonable to believe that increasing our understanding of CAFs inter- and intra-tumoral heterogeneity will be key to fully grasp CAFs secretome diverseness and potentiate anti-cancer therapy.

## Author Contributions

JL, JM-J, and AC participated to the manuscript preparation and revision (writing, reviewing, discussing, and editing). JB-R made substantial contribution to the manuscript revision. All listed authors approved this manuscript for publication.

## Conflict of Interest

The authors declare that the research was conducted in the absence of any commercial or financial relationships that could be construed as a potential conflict of interest.

## Abbreviations

AKT: protein kinase B
ATRA: all-trans retinoic acid
CCL: C–C motif chemokine
COX2: cyclooxygenase 2
CXC: C–X–C motif chemokine
CXCL: CXC ligand
CXCR: CXC receptor
DKK-1: Dickkopf-related protein 1
FGF: fibroblast growth factor
GDF-15: growth differentiation factor 15
GM-CSF: granulocyte-macrophage colony-stimulating factor
HER3: epidermal growth factor receptor 3
HGF: hepatocyte growth factor
HIF: hypoxia-inducible factor
HspA1A: heat shock 70 kDa protein 1A
ICAM-1: intercellular adhesion molecule 1
IFN: interferon
IGF: insulin-like growth factor
IGF1R: IGF 1 receptor
IGFBP: insulin-like growth factor-binding protein
JAK: Janus kinase
JNK: Jun N-terminal kinase
KLK: kallikrein
LIF: leukemia inhibitory factor
M-CSF: macrophage colony-stimulating factor
MAPK: mitogen-activated protein kinase
MCP-1: monocyte chemoattractant protein 1
MIF: macrophage migration inhibitory factor
MMP: matrix metallopeptidase
mTOR: mammalian target of rapamycin
MYH-11: myosin 11
NF-κB: nuclear factor-kappa B
NRG: neuregulin
OXPHOS: oxidative phosphorylation system
P38 MAPK: p38 mitogen-activated protein kinase
PDGF: platelet-derived growth factor
PDGFRB: platelet-derived growth factor receptor beta
PDPN: podoplanin
PGE2: prostaglandin E2
PI3K: phosphoinositide 3-kinase
POSTN: periostin
PAR: protease-activated receptor
RAGE: receptor for advanced glycation end products
miRNA: microRNA
mRNA: messenger RNA
lncRNA: long non-coding RNA
S100A11: S100 calcium-binding protein A11
SATB-1: special AT-rich sequence-binding protein-1
SDF: stromal cell-derived factor
SFRP4: secreted frizzled-related protein 4
Shp: Src homology 2-containing protein tyrosine phosphatase
SMAD: acronym for a signal transducer family
SOCS: suppressor of cytokine signaling 1
STAT: signal transducer and activator of transcription
TAZ: transcriptional coactivator with PDZ-binding motif, also known as Wwtr1
TET: ten-eleven translocation enzyme
THBS: thrombospondin
TIMP: tissue inhibitors of MMP
TNC: tenascin C
TNF: tumor necrosis factor
TPL2: tumor progression locus 2
VCAM: vascular cell adhesion protein
VEGF: vascular endothelial growth factor
VEGFA: VEGF type A
YAP: yes-associated protein.
